# Medical College Notes

**Published:** 1889-10

**Authors:** 


### Medical College Notes.

DR. JOHN A. MILLER, Professor of Medical Chemistry and Toxicology in the Medical Department of Niagara University, has received the appointment of Instructor in Quantitative Analysis in Cornell University. Dr. Miller has arranged his work at Cornell so as to be able to give his usual course of lectures here during the Winter.

---

THE MEDICAL DEPARTMENT of the University of Buffalo opened its forty-fourth year Monday evening, September 23, 1880, with a large class. The faculty is again changed, Dr. Witthaus's resignation having been accepted, and his place filled by Professor C. M. Hill, of Watertown.

The introductory lecture was given by Dr. James Wright Putnam, who took the occasion to place some of the principles of our profession before his audience. The fact that the profession is a liberal one not bound by the past to any isms or pathies, was strongly insisted upon, and the advantage of specialism pointed out.

THE Medical Department of Niagara University opened for the session of 1889–'90 on Wednesday evening, September 18th. The address was given by Professor George E. Fell. After the address, Professor John Cronyn, President of the Faculty, announced that before the beginning of another session the college would be greatly enlarged to accommodate the increased number of students.

